# Antimicrobial Stewardship Program: actions and education in Brazilian pediatric intensive care units*

**DOI:** 10.1590/1518-8345.7582.4656

**Published:** 2025-08-18

**Authors:** Eliane Carlosso Krummenauer, Mariana Portela de Assis, Mara Rubia Santos Gonçalves, Magda Machado de Miranda Costa, Rochele Mosmann Menezes, Fabio Araujo Motta, Jane Dagmar Pollo Renner, Marcelo Carneiro

**Affiliations:** 1 Universidade de Santa Cruz do Sul, Santa Cruz do Sul, RS, Brazil.; 2 Agência Nacional de Vigilância Sanitária, Brasília, DF, Brazil.; 3 Hospital Pequeno Príncipe, Curitiba, PR, Brazil.

**Keywords:** Antimicrobial Stewardship, Health Resources, Drug Resistance, Microbial, Education, Efficacy, Surveys and Questionnaires

## Abstract

to evaluate the educational elements and actions of the Antimicrobial Stewardship Program (ASP) in Pediatric Intensive Care Units (PED-ICU) in Brazil.

this was a multicenter, prospective study carried out between October 2022 and January 2023, using an electronic form. Educational actions for professionals and patients on microbial resistance were analyzed, as well as interventions to improve prescribing, such as prospective audits, feedback and pre-authorization. The data was grouped by location, care profile and number of beds and analyzed using parametric and non-parametric statistical methods.

of the 219 PED-ICUs with ASP in place, 44.7% were in the Southeast and 58.9% carried out continuing education for professionals. Only 6.8% promoted educational activities for patients and companions, concentrated in public hospitals with more than 200 beds. The education component showed weaknesses, especially in smaller hospitals, while actions to improve prescribing were more consolidated, with 70.8% implemented. Private hospitals and those in the Southeast and South stood out in terms of adherence to management practices.

the study revealed significant disparities in the implementation of ASPs in Brazilian PED-ICUs, highlighting the need to strengthen continuing education and engage all those involved in order to increase adherence, reduce antimicrobial resistance and improve pediatric care.

## Highlights

**(1)** ASP in Brazilian PED-ICU shows gaps in antimicrobial education and prescription. **(2)** Insufficient education compromises the rational use of antimicrobials in Brazil. **(3)** Regional inequality challenges ASP in public and private hospitals. **(4)** Larger, private hospitals lead the way in adherence to ASP in PED-ICU in Brazil. **(5)** Educational strategies and audits strengthen antimicrobial control in ASP.

## Introduction

Antimicrobial resistance (AMR) is a global threat to public health, especially in Latin America, where it has increased significantly in recent years^(1-2)^. One strategy to monitor this situation is to understand the development of Antimicrobial Stewardship Programs (ASP) and the barriers to their implementation, ensuring actions and funding for effective execution and program sustainability^(3-4)^.

The World Health Organization (WHO) has outlined a global action plan to address AMR, highlighting the fundamental role of Antimicrobial Stewardship Programs (ASP) in tackling this problem^(3)^.

In Brazil, efforts began in 1998, culminating in the drafting of national action plans against AMR and guidelines for implementing ASP. In 2019, the first national evaluation took place in hospitals with Adult Intensive Care Units (AD-ICU)^(5-8)^. This process was expanded with the second survey (2022/2023), which included hospitals with and without AD-ICUs and also evaluated Pediatric Intensive Care Units (PED-ICUs).

ASP is a strategy designed to implement measures to prevent harm related to the inappropriate use of antimicrobials, one of the main causes of drug harm in healthcare^(9)^. Improving clinical outcomes, such as morbidity and mortality, and optimizing costs are central objectives of the program^(3)^. To achieve this, a partnership between all the professionals involved is essential, based on positive behaviors and effective clinical leadership^(10-11)^.

The pediatric age group is increasingly exposed, especially in PED-ICUs, where there is a shortage of reliable and objective information on AMR and ASP in these care settings^(1,12-13)^.

There are significant knowledge gaps in antimicrobial stewardship, exacerbated by technical difficulties such as the shortage of qualified professionals and the absence of robust and systematic educational strategies for continuous training^(11,14-15)^. These gaps compromise the development and effective implementation of actions to improve antimicrobial prescribing, which require not only technical knowledge, but also the integration of aspects such as indication, prescription, dispensing, dilution, application, continuous reassessment and monitoring of adverse reactions and clinical evolution. These elements are crucial for ensuring the rational use of antimicrobials and for incorporating real data into institutional education and control programs. The lack of alignment between the stages of the drug cycle and institutional educational programs reflects structural and operational challenges that demand more consistent strategies for multi-professional training and for strengthening monitoring and auditing actions^(6)^.

It should also be noted that there are real difficulties in measuring the use of antimicrobials in pediatrics^(16)^. A useful form of evaluation, especially in hospitalized children, is the calculation of days of therapy (DOT) or days free of therapy (DFT)^(6,17)^. Understanding the evolution of AMR remains a global challenge, especially in pediatric care, which is often neglected.

The aim of this study was to evaluate the educational elements and actions of the Antimicrobial Stewardship Program (ASP) in Pediatric Intensive Care Units (PED-ICU) in Brazil.

## Method

### Study design

This is a prospective, cross-sectional, multicenter study.

### Setting

The study was carried out in hospitals with pediatric intensive care units (PED-ICU) located in different regions of Brazil, covering both public and private institutions.

### Period

The data was collected between October 2022 and January 2023.

### Population

According to data provided by the National Health Surveillance Agency (ANVISA), in 2022, Brazil had 662 hospitals with PED-ICU distributed among the regions. The Southeast region stood out with 346 hospitals (52.26%), followed by the Northeast with 110 (16.62%) and the South with 83 (12.54%). The study population was made up of Brazilian hospitals with PED-ICU, which had formalized ASP programs. A total of 393 PED-ICUs (59.36%) took part in the survey, of which 219 had ASP implemented and made up the sample for this study^(5-8)^.

### Selection criteria

The study included hospitals that had pediatric intensive care units (PED-ICU) and that had an ASP in place.

### Sample definition/Participants

Data was collected from participants in the second national ASP survey, coordinated by ANVISA/Ministry of Health, which included hospitals from all three spheres of health (federal, state and municipal). The survey was completed by the health services after ANVISA sent out emails inviting the institutions to take part in the study. Selection was based on voluntary adherence to completing the survey instrument by professionals representing the ASP. The sample consisted of 219 hospitals with PED-ICU, representing institutions of different sizes and regions of Brazil.

### Instrument used for data collection

Participation was voluntary, with professionals representing the ASP filling in an electronic form (Google Forms®). The data was obtained from a cross-section of information from a multicenter research database. A structured instrument was used, based on a questionnaire developed to assess ASP practices. The questionnaire was validated in advance and included questions about the study variables, distributed in sections covering education, antimicrobial prescribing actions and institutional characteristics^(18)^. The questionnaire was collected through an electronic system, ensuring greater reach and standardization of responses. The instrument allowed services to be classified as inadequate, basic, intermediate or advanced, based on a score assigned to each component assessed. There were 46 dichotomous questions (YES/NO) and 15 questions with multiple answers.

### Data collection

Data was collected between October 2022 and January 2023, using a validated instrument to assess the implementation of ASPs^(18)^.

### Data collection and study variables

For the purposes of this study, it was decided to cut out the data obtained and only hospitals with PED-ICU that had ASP implemented and component 3: education and component 4: development of actions to improve antimicrobial prescribing were examined. Component 3 assessed training actions for the institution’s professionals and patients on microbial resistance and antimicrobial use. Component 4 analyzed interventions such as prospective auditing, feedback and pre-authorization of antimicrobials^(6)^. In addition, the characteristics of these hospitals, such as location, care profile and size.

The hospitals were classified as Private: run by a private, charitable or philanthropic entity and Public: run by a municipal, state or federal government institution^(19)^. Conveniently, according to quantity, in number of care beds, up to 100 beds, 101/199 beds and ≥ 200 beds and according to Brazilian regions^(19)^.

### Data processing and analysis

All analyses were conducted using the Statistical Package for the Social Sciences (SPSS, v. 23, IBM, Armonk, NY). The comparison of the continuous scores for component 3: education and component 4: development of actions to improve antimicrobial prescribing in PED-ICU between the type of institution, the number of beds available and the region was carried out using two approaches: the first with parametric tests using the bootstrapping procedure with 5000 resamples and the Bias-corrected and accelerated (BCa) method; and the second with non-parametric tests due to the non-normal distribution of the variables.

The results were expressed as measures of central tendency (mean and median) and dispersion [standard deviation (SD) and interquartile range (IQR)], grouped by type of care and number of beds.

The comparison between the type of institution used Student’s t-tests for independent samples and the Mann-Whitney U-test, while the comparisons between the number of beds available and the region used one-way analysis of variance (ANOVA) and the Kruskal-Wallis test. In addition, a posteriori Games-Howell tests were used to identify which pairs differed, adjusting p-values according to the number of comparisons between groups^(20)^. Standardized differences were calculated using Cohen’s d effect size measure (d) to identify the magnitude of the difference. The d values were classified according to the cut-off points: d ≤ 0.49 = small difference; 0.50 ≤ d ≤ 0.79 = medium difference; and d ≥ 0.80 = large difference^(20)^.

Finally, comparisons of the relative frequencies of ‘yes’ answers between the type of institution, the number of beds available and the region were made using the a posteriori z-test in the chi-square analysis, adjusting p-values using the Bonferroni method, according to the number of comparisons between groups. Values of p ≤ 0.05 were considered statistically significant^(20)^.

### Ethical aspects

The study was approved by the Human Research Ethics Committee of the University of Santa Cruz do Sul (Certificate of Submission for Ethical Appraisal -CAAE: 57231722.3.1001.5343).

## Results

Of the 219 hospitals with PED-ICUs, 18 professional categories were identified as being part of the ASP teams, with emphasis on the infectious disease physician, the representative of the Hospital Infection Control Committee, the clinical pharmacist, the nurse and the representative of the Pharmacy and Therapeutics Committee.

The general level of adherence to Component 3, related to education, and component 4, which deals with the development of actions to improve antimicrobial prescribing in PED-ICU, was the same for all regions. Component 3 reached basic level, while component 4 reached advanced level, respectively, with an average of 155 (70.8%) consolidated actions.

Of the institutions responding to the study, 44.7% were located in the Southeast and 22.4% in the South. More than 60.0% of the respondents belonged to private hospitals, with the Southeast (44.4%) and the South (25.0%) standing out. With regard to the number of hospital beds, with the exception of the North, all the other regions had hospitals with more than 200 beds, with the highest proportion in the Southeast (43.8%). The data relating to these categorizations is shown in Table 1.

**Table 1-:** Absolute (n*) and relative (%^†^) characterization by region, type of hospital and number of beds of the 219 hospitals that had ASP^‡^ in Brazil, 2023 survey. Santa Cruz do Sul, RS, Brazil, 2023

	Type of hospital n* (% ^†^ )		Total n* (% ^†^ )	Number of beds n* (% ^†^ )		
	Private	Public		Up to 100 beds	101/199 beds	≥ 200 beds
North	12 (63.2)	7 (36.8)	19 (100)	6 (31.6)	9 (47.4)	4 (21.1)
Northeast	23 (63.9)	13 (36.1)	36 (100)	7 (19.4)	8 (22.2)	21 (58.3)
Mid-west	14 (82.4)	3 (17.6)	17 (100)	2 (11.8)	4 (23.5)	11 (64.7)
Southeast	71 (72.4)	27 (27.6)	98 (100)	22 (22.4)	27 (27.6)	49 (50.0)
South	40 (81.6)	9 (18.4)	49 (100)	4 (8.2)	18 (36.7)	27 (55.1)
Total n (%)	160 (100)	59 (100)	219 (100)	41 (100)	66 (100)	112 (100)

*n = Number; ^†^% = Percentage; ^‡^ASP = Antimicrobial Stewardship Program

Table 2 shows that there was no difference in the scores using the parametric tests with bootstrapping procedure (p > 0.05) between public and private hospitals, number of beds and regions by level of adherence to the ASP, in relation to Components 3 (education) and 4 (development of actions to improve antimicrobial prescribing), respectively.

**Table 2-:** Comparison of components 3 (education) and 4 (development of actions to improve antimicrobial prescribing), between the type of institution, number of beds and geographical region of the 219 hospitals that had ASP* in Brazil, 2023 survey. Santa Cruz do Sul, RS, Brazil, 2023

	n ^†^	Component 3		*P* ^||^	Component 4		*P* ^||^
		Mean ± SD ^‡^	Median (IIQ) ^§^		Mean ± SD ^‡^	Median (IIQ) ^§^	
Type of institution							
Private	160	37.44 ± 36.42	40 (0; 65)	0.536	252.97 ± 53.50	262.5 (231.25; 285)	0,510
Public	59	41.53 ± 43.49	45 (0; 70)		247.71 ± 51.80	255 (205; 290)	
Number of beds							
Up to 100	41	35.00 ± 33.37	40 (0;66.25)	0.768	241.10 ± 57.19	260 (225; 286.25)	0,276
101 to 199	66	39.77 ± 42.29	42.5 (0; 70)		249.24 ± 52.76	265 (235; 293.75)	
Over 200	112	39.11 ± 37.95	40 (0; 65)		256.74 ± 51.32	260 (207.5; 280)	
Region							
Mid-West	17	35.88 ± 40.59	35 (0; 62.5)	0.534	270.29 ± 35.11	275 (252.5; 290)	0,324
Northeast	36	38.47 ± 34.90	42.5 (0; 70)	0.534	250.69 ± 67.11	275 (211.25; 303.75)	0,324
North	19	59.21 ± 55.86	55 (0; 90)		251.05 ± 55.82	250 (225; 315)	
Southeast	98	35.31 ± 36.42	37.5 (0; 70)		250.56 ± 50.60	260 (223.75; 285)	
South	49	37.96 ± 34.82	45 (0; 60)		247.86 ± 50.71	260 (232.5; 280)	

*ASP = Antimicrobial Stewardship Program; ^†^n = Number; ^‡^DP = Standard Deviation; ^§^IIQ = Interquartile Range; ^||^P = Significance Level

The results indicate that private hospitals had averages of 37.44 ± 36.42 in Component 3 and 252.97 ± 53.50 in Component 4, while public hospitals had averages of 41.53 ± 43.49 and 247.71 ± 51.80, respectively. As for the number of beds, hospitals with up to 100 beds had averages of 35.00 ± 33.37 in Component 3 and 241.10 ± 57.19 in Component 4; those with 101 to 199 beds, 39.77 ± 42.29 and 249.24 ± 52.76; and those with more than 200 beds, 39.11 ± 37.95 and 256.74 ± 51.32, respectively. Regionally, the highest average in Component 4 was observed in the Midwest region (270.29 ± 35.11), while in Component 3 the average was 35.88 ± 40.59.

**Figure 1-:**
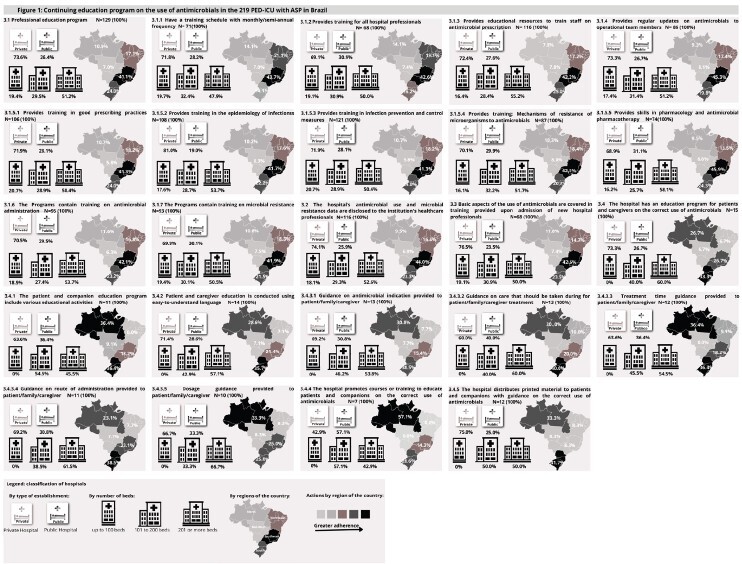
Comparison of the behavior of component 3 (education) between the type of institution, number of beds and region of the 219 hospitals that had ASP* in Brazil, according to the 2023 survey. Santa Cruz do Sul, RS, Brazil, 2023

Figure 1 shows the absolute frequencies of affirmative responses (YES) in the survey related to Component 3, which addresses education activities on the rational use of antimicrobials. These responses are distributed according to the type of hospital, number of beds and geographical regions. Of the 219 participating hospitals, 129 (58.9%) reported carrying out some educational activity on the subject. The results show that public hospitals had a higher absolute frequency of affirmative responses, especially in actions aimed at educating patients and carers about the rational use of antimicrobials.

The analysis by hospital size indicates that medium-sized hospitals, with 101 to 199 beds, showed greater adherence to educational activities compared to other size categories. Regionally, the North region had a higher frequency of activities related to antimicrobial education, especially public hospitals in this region. These results show the variation in adherence to educational practices between the different institutional and regional contexts, as detailed in Figure 1.

*ASP - Antimicrobial Stewardship Program

**Figure 2-:**
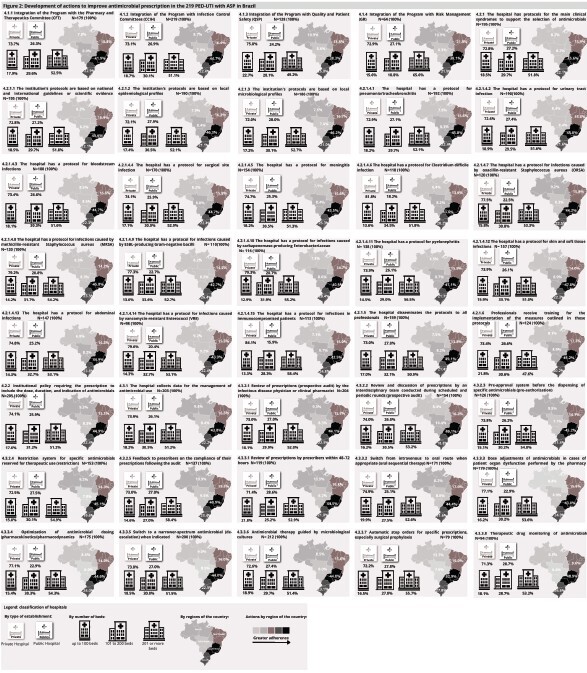
Comparison of the behavior of component 4 (development of actions to improve antimicrobial prescribing) between the type of institution, number of beds available and region of the 219 hospitals that had ASP* in Brazil, 2023 survey. Santa Cruz do Sul, RS, Brazil, 2023

Figure 2 shows the behavior of actions to improve antimicrobial prescribing (component 4). The institutions had 70.8% of consolidated actions. It was noted that the absolute frequency (YES response to the survey) stood out in private hospitals with more than 200 beds in the southeast of Brazil.

*ASP = Antimicrobial Stewardship Program

Private institutions showed greater adherence to actions related to Component 4, with emphasis on practices such as regular review of prescriptions, adoption of clinical decision support systems and implementation of institutional protocols. Public institutions, although with a lower overall frequency, stood out in specific actions, such as integration with hospital infection control committees and reviewing microbiological data to support prescriptions. In relation to the number of beds, hospitals with up to 100 beds showed lower overall adherence to Component 4 actions, while hospitals with 101 to 199 beds showed higher frequency in actions such as reviewing prescriptions and using microbiological data. Hospitals with more than 200 beds had the highest adherence, with structured practices such as integration with hospital infection control committees and regular review of prescriptions standing out.

Regionally, the greatest adherence to Component 4 actions was identified in the Southeast and South, with practices such as the implementation of evidence-based prescription protocols, review of prescriptions and integration with hospital infection control committees standing out. Despite this, relevant specific efforts were observed in the Midwest and North regions, highlighting the variability in the implementation of practices aimed at improving antimicrobial prescribing between the different regions of Brazil.

These results indicate that private institutions, larger hospitals and those in the southeast and south concentrate most actions aimed at developing practices to improve antimicrobial prescribing.

**Figure 3-:**
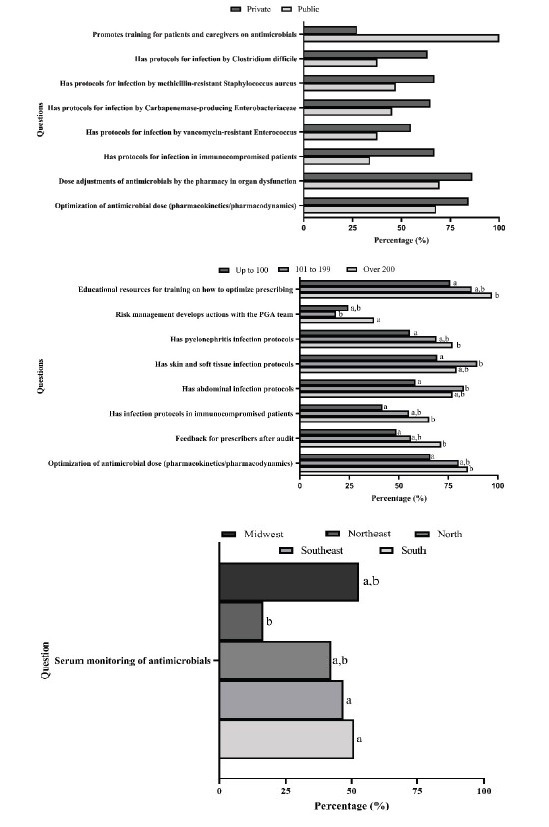
Comparison of the relative frequency of “yes” answers to the questions in components 3 (education) and 4 (development of actions to improve antimicrobial prescribing) between the type of institution, the number of beds available and question 4.3.3.8: Serum antimicrobial monitoring in component 4, between the regions of the 219 hospitals that had an ASP* in Brazil, 2023 survey. Santa Cruz do Sul, RS, Brazil, 2023

Note: Subscript letters indicate statistically significant differences (Bonferroni p < 0.05) in relative frequencies between groups; *ASP = Antimicrobial Stewardship Program

Figure 3 shows the comparisons between public and private hospitals for components 3 and 4 with p < 0.05. When comparing the overall scores for component 3: education and component 4: development of actions to improve antimicrobial prescribing in PED-ICUs, it was observed that there were no differences in the scores using parametric tests with bootstrapping procedure (all with p ≥ 0.276) between the type of institution, number of beds available and region. The non-parametric tests also found no differences in the comparisons (all with p ≥ 0.309). However, pairwise comparisons showed a statistically significant difference in the score for component 4 between the central-west and south-east regions [95% Confidence Interval (CI) of the difference: -38.04; -0.33; d = 0.41] and between the central-west and south (95% CI of the difference: -43.97; -0.42; d = 0.46).

## Discussion

This study represents the first national survey of ASP resources, evaluating the scenario of education and activities to improve antimicrobial prescribing in PED-ICUs in Brazil. The results indicated a variability in practices, highlighting weaknesses in the development of continuing education programs and regional and institutional disparities, especially between public and private hospitals. The predominance of private hospitals with more than 200 beds in the Southeast and South regions, which concentrate most of the country’s population and Gross Domestic Product (GDP), reflects historical inequalities in health infrastructure and resources^(21)^. This concentration contrasts with the higher relative number of hospitals with PED-ICU in the Northeast, but with lower adherence to completing the survey, and a lower frequency of hospitals with ASP in place in the North and Mid-West regions.

Formal ASP programs in public hospitals with up to 200 beds are limited, amplifying structural challenges already identified, such as quantifying the use of pediatric antimicrobials, defining efficacy measures and the lack of evidence-based local guidelines^(1,16,22)^. In line with this, a study in Latin America highlighted barriers such as lack of funding, scarcity of training opportunities during working hours and lack of topics applicable to the implementation of ASP and metrics for behavior change^(4)^. These obstacles, which are common in low- and middle-income countries, seem to be echoed in Brazil, where 40% of institutions reported having no educational activities on antimicrobials.

The comparison between Components 3 (education) and 4 (development of actions) revealed discrepancies. While Component 4 showed greater consolidation, with 70% of actions implemented, Component 3 showed significant gaps, especially in smaller and public hospitals. This finding is consistent with international studies, which have reported the absence of regular education programs in low- and middle-income countries^(17)^. Training professionals and continuing education are essential tools for increasing understanding of antimicrobial resistance (AMR) and promoting behavioral changes^(23-25)^. The introduction of innovative strategies, such as rapid antimicrobial approval systems via text messaging, has reduced costs and optimized drug use in recent studies^(26)^.

The results also highlighted that the implementation of evidence-based protocols, such as those for specific infectious syndromes, was present in 89% of institutions, but often without audits to ensure their application. The lack of practical guidelines for the rational use of antimicrobials was associated with increased consumption of broad-spectrum antibiotics, in line with studies that have identified a relationship between weak protocols and higher infant morbidity and mortality^(27-28)^.

The analysis in Figure 3 reinforces the importance of educational interventions and the harmonization of practices between different regions and types of hospitals. For example, serum antimicrobial monitoring showed statistically significant differences between the Midwest, Southeast and South regions, highlighting greater adherence in the more developed regions. This finding corroborates previous studies which have indicated the need for local adaptation in order to optimize the implementation of ASP^(29-39)^. In addition, feedback to prescribers after audits and optimization of antimicrobial doses (pharmacokinetics/pharmacodynamics) were more common in private hospitals with more than 200 beds, reinforcing the structural inequality between the public and private systems.

In this context, the strategic role of the nursing team stands out. As they are directly involved in critical stages of care, such as the administration, monitoring and reporting of antimicrobials, nursing staff should be the focus of permanent educational actions that increase their role, their technical qualifications and their clinical performance within the scope of ASPs. In addition, considering the centrality of nurses in the relationship with patients and their families, especially in pediatric units, it is essential that educational programs also include continuous guidance for companions, promoting the rational use of antimicrobials and strengthening adherence to practices to prevent microbial resistance.

At the same time, nursing record systems, which are often undervalued, need to be improved, computerized and integrated into the ASP, allowing for traceability of actions, support for clinical auditing and data feedback. This integration between multi-professional education, improving communication with users and strengthening records can make a decisive contribution to the sustainability and effectiveness of programs, especially in pediatric contexts that demand highly sensitive, family-centered care.

Limitations of the study include the risk of selection bias, due to the voluntary participation of hospitals, and differences in the frequency of responses between regions, which may not reflect the general panorama of Brazil. However, the study provides unpublished and relevant data to guide policies and institutional interventions that favor adherence to ASP, especially in pediatric settings. The integration of educational and infection control strategies with multi-professional support can contribute to reducing the overuse of antimicrobials and improving child morbidity and mortality indicators.

## Conclusion

The results of this survey highlight a significant imbalance between different hospital services in Brazil, including public and private hospitals, with up to 100 beds, between 101 and 199 beds and above 200 beds, in addition to regional disparities regarding educational resources available to professionals, patients and caregivers, as well as actions to improve antimicrobial prescribing in ASP. This variability reflects structural and geographic inequalities that require an integrated and collaborative national approach to combat AMR.

A key priority identified in this study was the urgent need for continuing education, both for health professionals and service users. Systematic, ongoing educational programs supported by management committed to ASP are essential to sustain behavioral changes and promote judicious use of antimicrobials. This educational gap is a critical challenge, especially in smaller hospitals, but also an opportunity for significant impact.

In this scenario, the need to reposition nursing as an agent that articulates care management, health education and patient safety is highlighted. The qualified inclusion of the nursing team in ASP can expand the scope of educational actions with professionals, patients and family members, in addition to contributing to the integration of clinical practices with registration, monitoring and auditing systems. Investing in the technical and relational training of these professionals, as well as in valuing their work in care records, represents a viable and high-impact strategy to consolidate the rational use of antimicrobials in critical pediatric settings. By strengthening these dimensions, Brazil will be able to not only reduce structural inequalities in the fight against microbial resistance, but also qualify care and promote sustainable advances in child health care. Given Brazil’s history of success in public health programs, such as the National Childhood Vaccination Program, it is possible to replicate similar strategies to strengthen adherence to ASP and reduce morbidity and mortality associated with AMR in PED-ICU. An essential next step will be to conduct cost-effectiveness analyses of the proposed interventions, including monitoring the profile of microorganisms causing infections and the use of antimicrobials, which will allow for more strategic and efficient planning.

Coordinated government and non-governmental efforts will be essential to address this challenge. These efforts should include expanding access to quality medicines, improving hospital and health infrastructure, expanding the logistics of clinical microbiology, and adequate monitoring of infections. In addition, professional organizations have an essential role to play in facilitating national and international collaboration networks to share good practices, train professionals, and support the implementation of strategies adapted to local realities.

This collaborative structure can increase the effectiveness of antimicrobial surveillance and use actions, strengthening antimicrobial resistance control indicators and reducing the global impact of AMR in children. Strengthening ASP in Brazil is not only a necessity, but an opportunity to promote sustainable and significant advances in child health and the safety of pediatric health services.

## References

[1] Antimicrobial Resistance Collaborators (2022). Global burden of bacterial antimicrobial resistance in 2019: a systematic analysis. Lancet.

[2] McMullan B., Bryant P. A., Duffy E., Bielicki J., De Cock P., Science M. (2023). Multinational consensus antimicrobial stewardship recommendations for children managed in hospital settings. Lancet Infect Dis.

[3] World Health Organization (2024). Antimicrobial resistance.

[4] Fabre V., Secaira C., Cosgrove S. E., Lessa F. C., Patel T. S., Alvarez A. A. (2023). Deep Dive Into Gaps and Barriers to Implementation of Antimicrobial Stewardship Programs in Hospitals in Latin America. Clin Infect Dis.

[5] Agência Nacional de Vigilância Sanitária (BR) (2017). Acesse Plano de Ação para controle da resistência microbiana.

[6] Agência Nacional de Vigilância Sanitária (BR) (2024). Diretriz Nacional para Elaboração de Programa de Gerenciamento do Uso de Antimicrobianos em Serviços de Saúde. Revisão 2023.

[7] Agência Nacional de Vigilância Sanitária (BR) (2019). Projeto Stewardship Brasil: avaliação nacional dos programas de gerenciamento do uso de antimicrobianos em unidade de terapia intensiva adulto dos hospitais brasileiros. https://antigo.anvisa.gov.br/documents/33852/271855/Projeto+Stewardship+Brasil/435012dc-4709-4796-ba78-a0235895d901?version=1.0.

[8] Menezes R. M., Gonçalves M. R. S., Costa M. M. M., Krummenauer E. C., Carneiro G. M., Reuter C. P. (2022). Antimicrobial Stewardship Programmes in Brazil: introductory analysis. Research, Society and Development.

[9] World Health Organization (2023). Global burden of preventable medication-related harm in health care: a systematic review. https://www.who.int/publications/i/item/9789240088887.

[10] Alzunitan M. A., Edmond M. B., Alsuhaibani M. A., Samuelson R. J., Schweizer M. L., Marra A. R. (2022). Positive deviance in infection prevention and control: A systematic literature review. Infect Control Hosp Epidemiol.

[11] Abo Y. N., Freyne B., Kululanga D., Bryant P. A. (2022). The Impact of Antimicrobial Stewardship in Children in Low- and Middle-income Countries: A Systematic Review. Pediatr Infect Dis J.

[12] Manice C. S., Muralidhar N., Campbell J. I., Nakamura M. M. (2024). Implementation and Perceived Effectiveness of Prospective Audit and Feedback and Preauthorization by US Pediatric Antimicrobial Stewardship Programs. J Pediatric Infect Dis Soc.

[13] Gidey K., Aregawi S. G., Hailu B. Y., Asgedom S. W., Niriayo Y. L. (2024). Antimicrobial Use-Related Problems Among Hospitalized Pediatric Patients: A Prospective Observational Study. Infect Drug Resist.

[14] Abraão L. M., Figueiredo R. M., Gusmão V. C. L., Félix A. M., Ciofi-Silva C. L., Padoveze M. C. (2023). Brazilian Nurses Network Tackling the Antimicrobial Resistance (REBRAN): bringing the role of nurses from the shadow to the light. Rev Esc Enferm USP.

[15] Camerini F. G., Cunha T. L., Fassarella C. S., Henrique D. M., Fortunato J. G. S. (2024). Nursing strategies in antimicrobial stewardship in the hospital environment: a qualitative systematic review. BMC Nurs.

[16] Cotton M. F., Sharland M. (2022). Antimicrobial Stewardship and Infection Prevention and Control in Low- and Middle-income Countries: Current Status and Best Practices. Pediatr Infect Dis J.

[17] Villanueva P., Coffin S. E., Mekasha A., McMullan B., Cotton M. F., Bryant P. A. (2022). Comparison of Antimicrobial Stewardship and Infection Prevention and Control Activities and Resources Between Low-/Middle- and High-income Countries. Pediatr Infect Dis J.

[18] Menezes R. M., Carneiro M., Gonçalves M. R. S., Costa M. M. M., Krummenauer E. C., Reuter C. P. (2022). Desenvolvimento e validação de questionário para autoavaliação dos programas de gerenciamento de antimicrobianos em unidade de terapia intensiva adulto. Rev Cient Multidiscip Núcleo Conhecimento.

[19] Portela M. C., Lima S. M., Barbosa P. R., Vasconcellos M. M., Ugá M. A., Gerschman S. (2004). Characterization of assistance among philanthropic hospitals in Brazil. Rev Saude Publica.

[20] Álvares M. (2019). Introdução à investigação quantitativa e análise SPSS.

[21] Instituto Brasileiro de Geografia e Estatística (2024). Cidades e Estados do Brasil.

[22] Rudnick W., Conly J., Thirion D. J. G., Choi K., Pelude L., Cayen J. (2023). Canadian Nosocomial Infection Surveillance Program. Antimicrobial use among paediatric inpatients at hospital sites within the Canadian Nosocomial Infection Surveillance Program, 2017/2018. Antimicrob Resist Infect Control.

[23] Sayood S., Bielicki J., Gandra S. (2024). Tackling inappropriate antibiotic use in low-and middle-income countries. Nat Med.

[24] Abuawad M., Ziyadeh-Isleem A., Mahamid A., Quzmar S., Ammar E., Shawahna R. (2024). Knowledge, perception, and attitudes of medical students towards antimicrobial resistance and stewardship: an observational cross-sectional study from Palestine. BMC Med Educ.

[25] Shitindi L., Issa O., Poyongo B. P., Horumpende P. G., Kagashe G. A., Sangeda R. Z. (2024). Comparison of knowledge, attitude, practice and predictors of self-medication with antibiotics among medical and non-medical students in Tanzania. Front Pharmacol.

[26] Agwu A. L., Lee C. K. K., Jain S. K., Murray K. L., Topolski J., Miller R. E. (2008). A World Wide Web-based antimicrobial stewardship program improves efficiency, communication, and user satisfaction and reduces cost in a tertiary care pediatric medical center. Clin Infect Dis.

[27] Ya K. Z., Win P. T. N., Bielicki J., Lambiris M., Fink G. (2023). Association Between Antimicrobial Stewardship Programs and Antibiotic Use Globally: A Systematic Review and Meta-Analysis. JAMA Netw Open.

[28] Reyes R. E., López M. J., Pérez J. E., Martínez G. (2023). Description of changes in clinical outcomes following the implementation of an antibiotic stewardship program in a level IV hospital. Biomedica.

[29] Harun G. D., Sumon S. A., Hasan I., Akther F. M., Islam M. S., Anwar M. U. (2024). Barriers, facilitators, perceptions and impact of interventions in implementing antimicrobial stewardship programs in hospitals of low-middle and middle countries: a scoping review. Antimicrob Resist Infect Control.

[30] Rolfe R., Kwobah C., Muro F., Ruwanpathirana A., Lyamuya F., Bodinayake C. (2021). Barriers to implementing antimicrobial stewardship programs in three low- and middle-income country tertiary care settings: findings from a multi-site qualitative study. Antimicrob Resist Infect Control.

[31] Fernandes T. B., Ramos S. F., Leitzke L. R. F., Alexandre R. G., Araújo J. M., Souza A. S. S. (2024). Use of antimicrobials in pediatric wards of five Brazilian hospitals. BMC Pediatr.

[32] Freudenhammer M., Hufnagel M., Steib-Bauert M., Mansmann U., De With K., Fellhauer M. (2023). Antibiotic use in pediatric acute care hospitals: an analysis of antibiotic consumption data from Germany, 2013-2020. Infection.

[33] Zaffagnini A., Rigotti E., Opri F., Opri R., Simiele G., Tebon M. (2024). Enforcing surveillance of antimicrobial resistance and antibiotic use to drive stewardship: experience in a paediatric setting. J Hosp Infect.

[34] Cusack R., Little E., Martin-Loeches I. (2024). Practical Lessons on Antimicrobial Therapy for Critically Ill Patients. Antibiotics (Basel).

[35] Muro F. J., Lyamuya F. S., Kwobah C., Bollinger J., Bodinayake C. K., Nagahawatte A. (2022). Opportunities for Improving Antimicrobial Stewardship: Findings From a Prospective, Multi-Center Study in Three Low- or Middle-Income Countries. Front Public Health.

[36] Setiawan E., Abdul-Aziz M. H., Roberts J. A., Cotta M. O. (2022). Hospital-Based Antimicrobial Stewardship Programs Used in Low- and Middle-Income Countries: A Scoping Review. Microb Drug Resist.

[37] Yock-Corrales A., Naranjo-Zuñiga G. (2023). Regional Perspective of Antimicrobial Stewardship Programs in Latin American Pediatric Emergency Departments. Antibiotics (Basel).

[38] Restrepo-Arbeláez N., Garcia-Betancur J. C., Pallares C. J., Villegas M. V. (2023). Antimicrobial Stewardship Programs in Latin America and the Caribbean: A Story of Perseverance, Challenges, and Goals. Antibiotics (Basel).

[39] Pallares C. J., Porras J., De La Cadena E., García-Betancur J. C., Restrepo-Arbeláez N., Viveros S. M. C. (2023). Antimicrobial stewardship programs in seven Latin American countries: facing the challenges. BMC Infect Dis.

