# Supplementary material for: Antimicrobial Stewardship Program: actions and education in Brazilian pediatric intensive care units
Source: Rev Lat Am Enfermagem. 2025 Aug 18;33:e4656. doi: 10.1590/1518-8345.7582.4656 (PMC12364454; doi:10.1590/1518-8345.7582.4656)
Supplement: Translation

# Translation: Programa de Gerenciamento de Antimicrobianos: ações e educação nas unidades de terapia intensiva pediátricas brasileiras*

**DOI:** 10.1590/1518-8345.7582.4657

**Authors:** Eliane Carlosso Krummenauer, Mariana Portela de Assis, Mara Rubia Santos Gonçalves, Magda Machado de Miranda Costa, Rochele Mosmann Menezes, Fabio Araujo Motta, Jane Dagmar Pollo Renner, Marcelo Carneiro

**Affiliations:** 1 Universidade de Santa Cruz do Sul, Santa Cruz do Sul, RS, Brasil.; 2 Agência Nacional de Vigilância Sanitária, Brasília, DF, Brasil.; 3 Hospital Pequeno Príncipe, Curitiba, PR, Brasil.

**Keywords:** Gestão de Antimicrobianos, Recursos em Saúde, Resistência Microbiana a Medicamentos, Educação, Eficácia, Inquéritos e Questionários

## Abstract

avaliar os elementos de educação e ações do Programa de Gerenciamento de Antimicrobianos (PGA) em Unidades de Terapia Intensiva Pediátrica (PED-UCI) no Brasil.

estudo multicêntrico, prospectivo, realizado entre outubro de 2022 e janeiro de 2023, com formulário eletrônico. Foram analisadas ações educativas para profissionais e pacientes sobre resistência microbiana, além de intervenções para melhorar a prescrição, como auditorias prospectivas, *feedback* e pré-autorização. Os dados foram agrupados por localização, perfil de atendimento e número de leitos e analisados com métodos estatísticos paramétricos e não paramétricos.

das 219 PED-UCI com PGA implementado, 44,7% estavam na região Sudeste e 58,9% realizavam educação permanente para profissionais. Apenas 6,8% promoviam atividades educativas para pacientes e acompanhantes, concentradas em hospitais públicos com mais de 200 leitos. O componente educação apresentou fragilidades, especialmente em hospitais menores, enquanto as ações para melhorar a prescrição foram mais consolidadas, com 70,8% de implementação. Hospitais privados e das regiões Sudeste e Sul destacaram-se na adesão às práticas de gerenciamento.

o estudo revelou disparidades significativas na implementação dos PGAs em PED-UCI brasileiras, evidenciando a necessidade de fortalecer a educação permanente e engajar todos os envolvidos, visando ampliar a adesão, reduzir a resistência antimicrobiana e melhorar o cuidado pediátrico.

## Destaques

**(1)** PGA em PED-UCI brasileiras mostra lacunas em educação e prescrição antimicrobiana. **(2)** Educação insuficiente compromete o uso racional de antimicrobianos no Brasil. **(3)** Desigualdade regional desafia o PGA em hospitais públicos e privados. **(4)** Hospitais maiores e privados lideram a adesão ao PGA em PED-UCI no Brasil. **(5)** Estratégias educativas e auditorias fortalecem o controle antimicrobiano no PGA.

## Introdução

A resistência aos antimicrobianos (AMR) é uma ameaça global à saúde pública, especialmente na América Latina, onde isso tem aumentado significativamente nos últimos anos^(1-2)^. Uma estratégia para acompanhar tal situação é compreender o desenvolvimento dos Programas de Gerenciamento de Antimicrobianos (PGA) e as barreiras à sua implementação, garantindo ações e financiamento para execução eficaz e sustentabilidade do programa^(3-4)^.

A Organização Mundial da Saúde (OMS) delineou um plano de ação global para abordar a AMR, destacando o papel fundamental dos *Antimicrobial Stewardship Programs* (ASP) no enfrentamento deste problema^(3)^.

No Brasil, os esforços começaram em 1998, culminando na elaboração de planos de ação nacional contra a AMR e diretrizes para implantação do PGA. Em 2019, ocorreu a primeira avaliação nacional em hospitais com Unidades de Cuidado Intensivo Adulto (AD-UCI)^(5-8)^. Esse processo foi ampliado com o segundo inquérito (2022/2023), que incluiu hospitais com e sem AD-UCI e avaliou também as Unidades de Cuidado Intensivo Pediátricas (PED-UCI).

O PGA é uma estratégia projetada para implementar medidas que previnam danos relacionados ao uso inadequado de antimicrobianos, uma das principais causas de danos medicamentosos na área da saúde^(9)^. Nesse sentido, melhorar os desfechos clínicos, como morbimortalidade, e otimizar os custos são objetivos centrais do programa^(3)^. Para isso, é fundamental a parceria entre todos os profissionais envolvidos, baseada em comportamentos positivos e liderança clínica eficaz^(10-11)^.

A faixa etária pediátrica está cada vez mais exposta, especialmente em PED-UCI, onde há escassez de informações confiáveis e objetivas sobre AMR e PGA, nos ambientes de cuidado^(1,12-13)^.

Existem lacunas significativas de conhecimento no manejo de antimicrobianos, agravadas por dificuldades técnicas, como a escassez de profissionais qualificados e a ausência de estratégias educativas robustas e sistemáticas para capacitação contínua^(11,14-15)^. As lacunas comprometem o desenvolvimento e a implementação eficaz de ações para melhorar a prescrição antimicrobiana, que exigem não apenas conhecimento técnico, mas também a integração de aspectos como indicação, prescrição, dispensação, diluição, aplicação, reavaliação contínua e monitoramento de reações adversas e evolução clínica. Tais elementos são cruciais para garantir o uso racional de antimicrobianos e para a incorporação de dados reais em programas institucionais de educação e controle. A falta de alinhamento entre as etapas do ciclo do medicamento e os programas educativos institucionais reflete desafios estruturais e operacionais que demandam estratégias mais consistentes para a capacitação multiprofissional e para o fortalecimento das ações de monitoramento e auditoria^(6)^.

Salienta-se, ainda, que existe uma dificuldade real em mensurar o uso de antimicrobianos na pediatria^(16)^. Uma forma útil de avaliação, especialmente em crianças hospitalizadas, é o cálculo de dias de terapia (DOT) ou dias livres de terapia (AFD)^(6,17)^. Compreender a evolução da AMR continua sendo um desafio global, especialmente no cuidado pediátrico, frequentemente negligenciado.

O objetivo deste estudo foi avaliar os elementos de educação e ações do Programa de Gerenciamento de Antimicrobianos (PGA) em Unidades de Terapia Intensiva Pediátrica (PED-UCI), no Brasil.

## Método

### Design do estudo

Trata-se de um estudo prospectivo, transversal e multicêntrico.

### Local

O estudo foi realizado em hospitais com unidades de terapia intensiva pediátrica (PED-UCI) localizados em diferentes regiões do Brasil, abrangendo instituições públicas e privadas.

### Período

Os dados foram coletados entre outubro de 2022 e janeiro de 2023.

### População

De acordo com dados disponibilizados pela Agência Nacional de Vigilância Sanitária (ANVISA), em 2022, o Brasil contava com 662 hospitais com PED-UCI distribuídas entre as regiões. A região Sudeste se destacou com 346 hospitais (52,26%), seguida pelas regiões Nordeste com 110 (16,62%) e Sul com 83 (12,54%). A população do estudo foi composta por hospitais brasileiros com PED-UCI, que tinham programas formalizados de PGA. Participaram do inquérito 393 PED-UCI (59,36%), das quais 219 apresentaram PGA implementados e compuseram a amostra deste estudo^(5-8)^.

### Critério de seleção

Foram incluídos no estudo os hospitais que possuíam unidades de terapia intensiva pediátrica (PED-UCI), e que tinham PGA implantado.

### Definição da amostra/Participantes

Foram recrutados os dados dos participantes do segundo inquérito nacional de PGA, coordenado pela ANVISA/Ministério da Saúde, que incluiu hospitais das três esferas de saúde (federal, estadual e municipal). O preenchimento do inquérito foi realizado pelos serviços de saúde após o envio de e-mails pela ANVISA, convidando as instituições a participarem do estudo. A seleção foi baseada na adesão voluntária ao preenchimento do instrumento de pesquisa pelos profissionais que representam o PGA. A amostra foi composta por 219 hospitais com PED-UCI, representando instituições de diferentes portes e regiões do Brasil.

### Instrumento utilizado para a coleta de dados

A participação foi voluntária, com preenchimento de um formulário eletrônico (*Google Forms*®) pelos profissionais representantes do PGA. Os dados foram obtidos a partir de um recorte de informações de um banco de dados de uma pesquisa multicêntrica. Foi utilizado um instrumento estruturado, fundamentado em um questionário desenvolvido para a avaliação das práticas do PGA. O questionário foi validado previamente e incluiu questões sobre as variáveis do estudo, distribuídas em seções que abordavam educação, ações de prescrição antimicrobiana e características institucionais^(18)^. A coleta foi realizada por meio de um sistema eletrônico, garantindo maior alcance e padronização das respostas. O instrumento permitiu a classificação dos serviços em inadequado, básico, intermediário ou avançado, com base em uma pontuação atribuída a cada componente avaliado. Foram aplicadas 46 questões dicotômicas (SIM/NÃO) e 15 questões com múltiplas respostas.

### Coleta de dados

Os dados foram coletados entre outubro de 2022 e janeiro de 2023, utilizando um instrumento validado para avaliar a implementação dos PGAs^(18)^.

### Coleta de dados e variáveis do estudo

Para fins deste estudo, optou-se por realizar um recorte nos dados obtidos e foram examinados somente os hospitais com PED-UCI que tinham PGA implementado e os componentes 3: educação e 4: desenvolvimento de ações para melhorar a prescrição de antimicrobianos. O componente 3 avaliou as ações de treinamento para os profissionais da instituição e pacientes sobre resistência microbiana e uso de antimicrobianos. Já o componente 4 analisou as intervenções como a auditoria prospectiva, *feedback* e pré-autorização de antimicrobianos^(6)^. Além disso, observou-se as características desses hospitais, como localização, perfil de atendimento e tamanho.

Os hospitais foram classificados em Privado: isto é, administrado por entidade particular, beneficente ou filantrópica, e Público: administrado por entidade governamental municipal, estadual ou federal^(19)^. De forma conveniente, conforme quantitativo, estabeleceu-se as categorias segundo o número de leitos de atendimento, sendo a primeira até 100 leitos; seguida de 101 a 199 leitos e, por fim, ≥ 200 leitos, de acordo com as regiões brasileiras^(19)^.

### Tratamento e análise dos dados

Todas as análises foram conduzidas utilizando o *Statistical Package for the Social Sciences* (SPSS, v. 23, IBM, Armonk, NY). A comparação das pontuações contínuas dos componentes 3: educação e 4: desenvolvimento de ações para melhorar a prescrição de antimicrobianos em PED-UCI deu-se considerando o tipo de instituição, o número de leitos disponíveis e a região, sendo realizada por meio de duas abordagens: a primeira com testes paramétricos, cujo procedimento de *bootstrapping* incluiu 5000 reamostragens e o método corrigido e acelerado por viés (BCa - *Bias-corrected and accelerated*). Enquanto isso, a segunda abordagem diz respeito a testes não paramétricos devido à distribuição não normal das variáveis.

Os resultados foram expressos em medidas de tendência central (média e mediana) e dispersão [desvio-padrão (DP) e intervalo interquartílico (IIQ) ], agrupados por tipo de atendimento e número de leitos.

A comparação entre o tipo de instituição utilizou os testes t de Student para amostras independentes e U de Mann-Whitney, enquanto isso, as comparações entre número de leitos disponíveis e região utilizaram a análise de variância (ANOVA) de um fator e o teste de Kruskal-Wallis. Ademais, testes *a posteriori* de Games-Howell foram utilizados para identificar quais pares diferiram, ajustando valores de p conforme o número de comparações entre grupos^(20)^. Diferenças padronizadas foram calculadas utilizando a medida de tamanho de efeito d de Cohen (d) para identificar a magnitude da diferença. Os valores de d foram classificados de acordo com os pontos de corte: d ≤ 0,49 = diferença pequena; 0,50 ≤ d ≤ 0,79 = diferença média; e d ≥ 0,80 = diferença grande^(20)^.

Por fim, comparações das frequências relativas de respostas ‘sim’ entre o tipo de instituição, o número de leitos disponíveis e a região foram realizadas utilizando o teste z a posteriori na análise de qui-quadrado, ajustando valores de p pelo método de Bonferroni, conforme o número de comparações entre grupos. Valores de p ≤ 0,05 foram considerados estatisticamente significativos^(20)^.

### Aspectos éticos

O estudo foi aprovado pelo comitê de ética em pesquisa com seres humanos da Universidade de Santa Cruz do Sul (Certificado de Apresentação para Apreciação Ética -CAAE: 57231722.3.1001.5343).

## Resultados

Dos 219 hospitais com PED-UCI, foram identificadas 18 categorias profissionais que compõem as equipes do PGA, com destaque para médico infectologista, representante da Comissão de Controle de Infecção Hospitalar, farmacêutico clínico, enfermeiro e representante da Comissão de Farmácia e Terapêutica.

O nível de adesão geral do componente 3, relacionado à educação, e do componente 4, que aborda o desenvolvimento de ações para melhorar a prescrição de antimicrobianos em PED-UCI, foi o mesmo para todas as regiões. O componente 3 obteve nível básico, enquanto o componente 4 alcançou nível avançado, respectivamente, com uma média de 155 (70,8%) de ações consolidadas.

Das instituições respondentes do estudo, 44,7% estavam localizadas na região Sudeste e 22,4% na região Sul. Mais de 60,0% dos respondentes pertenciam a hospitais privados, com destaque para a região Sudeste (44,4%) e a região Sul (25,0%). Em relação à quantidade de leitos hospitalares, com exceção da região Norte, todas as demais apresentaram hospitais com mais de 200 leitos, sendo a maior proporção observada na região Sudeste (43,8%). Os dados referentes a estas categorizações estão apresentados na Tabela 1.

**Tabela 1-:** Caracterização absoluta (n*) e relativa (%^†^) por regiões, tipo de hospital e número de leitos dos 219 hospitais que tinham PGA^‡^ no Brasil, inquérito de 2023. Santa Cruz do Sul, RS, Brasil, 2023

	Tipo de hospital n* (% ^†^ )		Total n* (% ^†^ )	Número de leitos n* (% ^†^ )		
	Privado	Público		Até 100 leitos	101/199 leitos	≥ 200 leitos
Norte	12 (63,2)	7 (36,8)	19 (100)	6 (31,6)	9 (47,4)	4 (21,1)
Nordeste	23 (63,9)	13 (36,1)	36 (100)	7 (19,4)	8 (22,2)	21 (58,3)
Centro-Oeste	14 (82,4)	3 (17,6)	17 (100)	2 (11,8)	4 (23,5)	11 (64,7)
Sudeste	71 (72,4)	27 (27,6)	98 (100)	22 (22,4)	27 (27,6)	49 (50,0)
Sul	40 (81,6)	9 (18,4)	49 (100)	4 (8,2)	18 (36,7)	27 (55,1)
Total n (%)	160 (100)	59 (100)	219 (100)	41 (100)	66 (100)	112 (100)

*n = Número; ^†^% = Percentual; ^‡^PGA = Programa de Gerenciamento de Antimicrobianos

A Tabela 2 mostra que não houve diferença nas pontuações por meio dos testes paramétricos com procedimento *bootstrapping* (p > 0,05) entre hospitais públicos e privados, número de leitos e regiões por nível de adesão ao PGA, em relação aos Componentes 3 (educação) e 4 (desenvolvimento de ações para melhorar a prescrição de antimicrobianos), respectivamente.

**Tabela 2-:** Comparação dos componentes 3 (educação) e 4 (desenvolvimento de ações para melhorar a prescrição de antimicrobianos), entre o tipo de instituição, número de leitos e região geográfica dos 219 hospitais que tinham PGA* no Brasil, inquérito de 2023. Santa Cruz do Sul, RS, Brasil, 2023

	n ^†^	Componente 3		*P* ^||^	Componente 4		*P* ^||^
		Média ± DP ^‡^	Mediana (IIQ) ^§^		Média ± DP ^‡^	Mediana (IIQ) ^§^	
Tipo de instituição							
Privada	160	37,44 ± 36,42	40 (0; 65)	0,536	252,97 ± 53,50	262,5 (231,25; 285)	0,510
Pública	59	41,53 ± 43,49	45 (0; 70)		247,71 ± 51,80	255 (205; 290)	
Número de leitos							
Até 100	41	35,00 ± 33,37	40 (0;66,25)	0,768	241,10 ± 57,19	260 (225; 286,25)	0,276
101 a 199	66	39,77 ± 42,29	42,5 (0; 70)		249,24 ± 52,76	265 (235; 293,75)	
Mais de 200	112	39,11 ± 37,95	40 (0; 65)		256,74 ± 51,32	260 (207,5; 280)	
Região							
Centro-Oeste	17	35,88 ± 40,59	35 (0; 62,5)	0,534	270,29 ± 35,11	275 (252,5; 290)	0,324
Nordeste	36	38,47 ± 34,90	42,5 (0; 70)		250,69 ± 67,11	275 (211,25; 303,75)	
Norte	19	59,21 ± 55,86	55 (0; 90)		251,05 ± 55,82	250 (225; 315)	
Sudeste	98	35,31 ± 36,42	37,5 (0; 70)		250,56 ± 50,60	260 (223,75; 285)	
Sul	49	37,96 ± 34,82	45 (0; 60)		247,86 ± 50,71	260 (232,5; 280)	

*PGA = Programa de Gerenciamento de Antimicrobianos; ^†^n = Número; ^‡^DP = Desvio-padrão; ^§^IIQ = Intervalo Interquartílico; ^||^P = Nível de significância

Os resultados indicam que hospitais privados obtiveram médias de 37,44 ± 36,42 no Componente 3 e 252,97 ± 53,50 no Componente 4, enquanto os públicos apresentaram médias de 41,53 ± 43,49 e 247,71 ± 51,80, respectivamente. Quanto ao número de leitos, hospitais com até 100 leitos apresentaram médias de 35,00 ± 33,37 no Componente 3 e 241,10 ± 57,19 no Componente 4; aqueles com 101 a 199 leitos, 39,77 ± 42,29 e 249,24 ± 52,76; e os com mais de 200 leitos, 39,11 ± 37,95 e 256,74 ± 51,32, respectivamente. Regionalmente, a maior média no Componente 4 foi observada na região Centro-Oeste (270,29 ± 35,11), enquanto no Componente 3 a média foi de 35,88 ± 40,59.

A Figura 1 apresenta as frequências absolutas de respostas afirmativas (SIM) no inquérito relacionado ao Componente 3, que aborda atividades de educação sobre o uso racional de antimicrobianos. Essas respostas estão distribuídas conforme o tipo de hospital, o número de leitos e regiões geográficas. Dos 219 hospitais participantes, 129 (58,9%) relataram a realização de alguma atividade educativa voltada ao tema. Os resultados destacam que hospitais públicos tiveram maior frequência absoluta de respostas afirmativas, especialmente em ações direcionadas à educação de pacientes e acompanhantes sobre o uso racional de antimicrobianos.

A análise por porte hospitalar indica que hospitais de médio porte, com 101 a 199 leitos, apresentaram maior adesão às atividades educativas em comparação a outras categorias de porte. Regionalmente, o Norte obteve maior frequência de atividades relacionadas à educação sobre antimicrobianos, com destaque para hospitais públicos dessa região. Os resultados evidenciam a variação na adesão às práticas educativas entre os diferentes contextos institucionais e regionais, conforme detalhado na Figura 1.

**Figura 1-:** Comparação do comportamento do componente 3 (educação) entre o tipo de instituição, número de leitos e região dos 219 hospitais que tinham PGA* no Brasil, segundo inquérito de 2023. Santa Cruz do Sul, RS, Brasil, 2023

*PGA = Programa de Gerenciamento de Antimicrobianos

A Figura 2 expressa o comportamento das ações para melhorar a prescrição de antimicrobianos (componente 4). As instituições apresentavam 70,8% de ações consolidadas. Percebeu-se que a frequência absoluta (resposta SIM do inquérito) teve destaque nos hospitais privados, com mais de 200 leitos, sobretudo da região sudeste do Brasil.

**Figura 2-:** Comparação do comportamento do componente 4 (desenvolvimento de ações para melhorar a prescrição de antimicrobianos) entre o tipo de instituição, número de leitos disponíveis e região dos 219 hospitais que tinham PGA* no Brasil, inquérito de 2023. Santa Cruz do Sul, RS, Brasil, 2023

*PGA = Programa de Gerenciamento de Antimicrobianos

As instituições privadas apresentaram maior adesão às ações relacionadas ao Componente 4, com destaque para práticas como a revisão regular de prescrições, adoção de sistemas de apoio à tomada de decisão clínica e implementação de protocolos institucionais. As instituições públicas, embora com menor frequência geral, destacaram-se em ações específicas, como integração com comissões de controle de infecção hospitalar e revisão de dados microbiológicos para suporte à prescrição. Em relação ao número de leitos, hospitais com até 100 leitos apresentaram menor adesão geral às ações do Componente 4, enquanto os hospitais com 101 a 199 leitos demonstraram maior frequência em ações como revisão de prescrições e uso de dados microbiológicos. Os hospitais com mais de 200 leitos registraram a maior adesão, destacando-se por práticas estruturadas, como integração com comissões de controle de infecção hospitalar e revisão regular de prescrições.

Regionalmente, a maior adesão às ações do Componente 4 foi identificada nas regiões Sudeste e Sul, com destaque para práticas como a implementação de protocolos de prescrição baseados em evidências, revisão de prescrições e integração com comissões de controle de infecção hospitalar. Apesar disso, esforços específicos relevantes foram observados nas regiões Centro-Oeste e Norte, evidenciando a variabilidade na implementação de práticas voltadas para a melhoria da prescrição de antimicrobianos entre as diferentes regiões do Brasil.

Esses resultados indicam que as instituições privadas, hospitais de maior porte e das regiões sudeste e sul concentram a maioria das ações voltadas ao desenvolvimento de práticas para melhorar a prescrição de antimicrobianos.

A Figura 3 mostra as comparações entre hospitais públicos e privados dos componentes 3 e 4 com p < 0,05. Na comparação das pontuações gerais do Componente 3: educação e do Componente 4: desenvolvimento de ações para melhorar a prescrição de antimicrobianos em PED-UCI, observou-se que não havia diferenças nas pontuações por meio dos testes paramétricos com procedimento *bootstrapping* (todos com p ≥ 0,276) entre o tipo de instituição, número de leitos disponíveis e região. Os testes não paramétricos também não identificaram diferenças nas comparações (todos com p ≥ 0,309). Todavia, as comparações feitas par a par demonstraram uma diferença estatisticamente significativa na pontuação do componente 4 entre as regiões centro-oeste e sudeste [Intervalo de Confiança (IC) 95% da diferença: -38,04; -0,33; d = 0,41] e entre centro-oeste e sul (IC95% da diferença: -43,97; -0,42; d = 0,46).

**Figura 3-:** Comparação da frequência relativa de respostas “sim” às questões dos componentes 3 (educação) e 4 (desenvolvimento de ações para melhorar a prescrição de antimicrobianos) entre o tipo de instituição, entre o número de leitos disponíveis e a questão 4.3.3.8: Monitorização sérica dos antimicrobianos do componente 4, entre as regiões, dos 219 hospitais que tinham PGA* no Brasil, inquérito de 2023. Santa Cruz do Sul, RS, Brasil, 2023

Nota: Letras subscritas indicam diferenças estatisticamente significativas (p de Bonferroni < 0,05) de frequências relativas entre grupos; *PGA = Programa de Gerenciamento de Antimicrobianos

## Discussão

Este estudo representa a primeira pesquisa nacional sobre os recursos em PGA, avaliando o cenário de educação e atividades para melhorar a prescrição de antimicrobianos em PED-UCI no Brasil. Os resultados indicaram uma variabilidade nas práticas, evidenciando fragilidades no desenvolvimento de programas de educação permanente e disparidades regionais e institucionais, especialmente entre hospitais públicos e privados. A predominância de hospitais privados, com mais de 200 leitos nas regiões Sudeste e Sul, que concentram a maior parte da população e do Produto Interno Bruto (PIB) do país, reflete desigualdades históricas em infraestrutura e recursos de saúde^(21)^. A concentração contrasta com a maior quantidade relativa de hospitais com PED-UCI no Nordeste, mas com menor adesão ao preenchimento do inquérito, e uma menor frequência de hospitais com PGA implantado nas regiões Norte e Centro-Oeste.

Programas formais de PGA em hospitais públicos e com até 200 leitos são limitados, amplificando desafios estruturais já identificados, como a quantificação do uso de antimicrobianos pediátricos, a definição de medidas de eficácia e a falta de diretrizes locais baseadas em evidências^(1,16,22)^. Em alinhamento, um estudo na América Latina destacou barreiras como a ausência de financiamento, escassez de oportunidades de treinamento durante a jornada de trabalho e carência de temas aplicáveis à implementação de ASP e métricas para mudança de comportamento^(4)^. Esses obstáculos, comuns em países de baixa e média renda, parecem ecoar no panorama brasileiro, onde 40% das instituições relataram não possuir atividades educativas sobre antimicrobianos.

A comparação entre os Componentes 3 (educação) e 4 (desenvolvimento de ações) revelou discrepâncias. Enquanto o Componente 4 apresentou maior consolidação, com 70% das ações implementadas, o Componente 3 mostrou lacunas significativas, especialmente em hospitais menores e públicos. Tal achado é consistente com estudos internacionais, que relataram a ausência de programas regulares de educação em países de baixa e média renda^(17)^. A formação de profissionais e a educação permanente são ferramentas essenciais para aumentar a compreensão da resistência antimicrobiana (AMR) e promover mudanças comportamentais^(23-25)^. A introdução de estratégias inovadoras, como sistemas rápidos de aprovação de antimicrobianos por mensagens de texto, reduziu custos e otimizou o uso de medicamentos em estudos recentes^(26)^.

Os resultados também destacaram que a implementação de protocolos baseados em evidências, como os para síndromes infecciosas específicas, estava presente em 89% das instituições, mas frequentemente sem auditorias que assegurem sua aplicação. A inexistência de diretrizes práticas para o uso racional de antimicrobianos foi associada ao aumento do consumo de antibióticos de amplo espectro, em linha com estudos que identificaram uma relação entre protocolos frágeis e maior morbimortalidade infantil^(27-28)^.

A análise da Figura 3 reforça a importância de intervenções educativas e da harmonização de práticas entre diferentes regiões e tipos de hospitais. Por exemplo, a monitorização sérica de antimicrobianos mostrou diferenças estatisticamente significativas entre as regiões Centro-Oeste, Sudeste e Sul, destacando maior adesão nas regiões mais desenvolvidas. Este achado corrobora estudos anteriores que indicaram a necessidade de adaptação local para otimizar a implementação de programas de PGA^(29-39)^. Além disso, a retroalimentação para prescritores após auditorias e a otimização de doses de antimicrobianos (farmacocinética/farmacodinâmica) foram mais comuns em hospitais privados com mais de 200 leitos, reforçando a desigualdade estrutural nos sistemas público e privado.

Nesse contexto, destaca-se o papel estratégico da equipe de enfermagem. Por estar diretamente envolvida em etapas críticas do cuidado, como administração, monitoramento e notificação de antimicrobianos, a enfermagem deve ser foco de ações educativas permanentes, que ampliem seu protagonismo, sua qualificação técnica e sua atuação clínica no âmbito dos PGAs. Além disso, considerando a centralidade do enfermeiro na relação com os pacientes e seus familiares, especialmente em unidades pediátricas, é essencial que programas educativos também contemplem a orientação contínua aos acompanhantes, promovendo o uso racional de antimicrobianos e fortalecendo a adesão às práticas de prevenção da resistência microbiana.

Paralelamente, os sistemas de registro de enfermagem, muitas vezes subvalorizados, precisam ser aprimorados, informatizados e integrados ao PGA, permitindo rastreabilidade das ações, suporte à auditoria clínica e retroalimentação de dados. Essa integração entre educação multiprofissional, qualificação da comunicação com os usuários e fortalecimento dos registros pode contribuir decisivamente para a sustentabilidade e eficácia dos programas, especialmente em contextos pediátricos que demandam cuidado altamente sensível e centrado na família.

Limitações do estudo incluem o risco de viés de seleção, devido à participação voluntária dos hospitais, e diferenças na frequência de respostas entre as regiões, o que pode não refletir o panorama geral do Brasil. Contudo, o estudo fornece dados inéditos e relevantes para guiar políticas e intervenções institucionais que favoreçam a adesão ao PGA, principalmente em cenários pediátricos. A integração de estratégias educativas e de controle de infecções com apoio multiprofissional pode contribuir para a redução do uso excessivo de antimicrobianos e a melhoria dos indicadores de morbimortalidade infantil.

## Conclusão

Os resultados deste inquérito destacam um desequilíbrio significativo entre os diferentes serviços hospitalares no Brasil, incluindo hospitais públicos e privados, com até 100 leitos, entre 101 a 199 leitos e acima de 200 leitos, além das disparidades regionais em relação aos recursos educacionais disponíveis para profissionais, pacientes e acompanhantes, bem como ações para melhorar a prescrição de antimicrobianos em PGA. Essa variabilidade reflete desigualdades estruturais e geográficas que exigem uma abordagem nacional integrada e colaborativa para combater a AMR.

Uma das principais prioridades identificadas neste estudo foi a necessidade urgente de educação permanente tanto para os profissionais de saúde quanto para os usuários do serviço. Programas educativos sistemáticos, contínuos e apoiados por gestões comprometidas com o PGA são fundamentais para sustentar mudanças comportamentais e promover a utilização criteriosa de antimicrobianos. Essa lacuna educacional é um desafio crítico, especialmente em hospitais menores, mas também uma oportunidade de impacto significativo.

Nesse cenário, destaca-se a necessidade de reposicionar a enfermagem como agente articulador entre a gestão do cuidado, a educação em saúde e a segurança do paciente. A inserção qualificada da equipe de enfermagem nos PGA pode ampliar o alcance das ações educativas junto a profissionais, pacientes e familiares, além de contribuir para a integração das práticas clínicas aos sistemas de registro, monitoramento e auditoria. Investir na capacitação técnica e relacional desses profissionais, assim como na valorização de sua atuação nos registros assistenciais representa uma estratégia viável e de alto impacto para consolidar o uso racional de antimicrobianos em ambientes pediátricos críticos. Ao fortalecer essas dimensões, o Brasil poderá não apenas reduzir desigualdades estruturais no enfrentamento da resistência microbiana, mas também qualificar o cuidado e promover avanços sustentáveis na atenção à saúde infantil.

Dado o histórico de sucesso do Brasil em programas de saúde pública, como o Programa Nacional de Vacinação Infantil, é possível replicar estratégias semelhantes para fortalecer a adesão ao PGA e reduzir a morbimortalidade associada à AMR em PED-UCI. Um próximo passo essencial será a realização de análises custo-eficácia das intervenções propostas, incluindo o monitoramento do perfil de microrganismos causadores de infecções e do uso de antimicrobianos, o que permitirá um planejamento mais estratégico e eficiente.

Esforços governamentais e não-governamentais coordenados serão indispensáveis para enfrentar esse desafio. Eles devem incluir a ampliação do acesso a medicamentos de qualidade, à melhoria da infraestrutura hospitalar e sanitária, à expansão logística da microbiologia clínica e o monitoramento adequado de infecções. Além disso, as organizações de classe têm um papel essencial ao facilitar redes de colaboração nacionais e internacionais para compartilhar boas práticas, capacitar profissionais e apoiar a implementação de estratégias adaptadas às realidades locais.

Portanto, a estrutura colaborativa pode aumentar a eficácia das ações de vigilância e utilização de antimicrobianos, fortalecendo os indicadores de controle de resistência antimicrobiana e reduzindo o impacto global da AMR em crianças. O fortalecimento do PGA no Brasil não é apenas uma necessidade, mas uma oportunidade de promover avanços sustentáveis e significativos na saúde infantil e na segurança dos serviços de saúde pediátrica.

# Translation: Programa de Gestión de Antimicrobianos: acciones y educación en las unidades de terapia intensiva pediátricas brasileñas*

**DOI:** 10.1590/1518-8345.7582.4655

**Authors:** Eliane Carlosso Krummenauer, Mariana Portela de Assis, Mara Rubia Santos Gonçalves, Magda Machado de Miranda Costa, Rochele Mosmann Menezes, Fabio Araujo Motta, Jane Dagmar Pollo Renner, Marcelo Carneiro

**Affiliations:** 1 Universidade de Santa Cruz do Sul, Santa Cruz do Sul, RS, Brasil.; 2 Agência Nacional de Vigilância Sanitária, Brasília, DF, Brasil.; 3 Hospital Pequeno Príncipe, Curitiba, PR, Brasil.

**Keywords:** Programas de Optimización del Uso de los Antimicrobianos, Recursos en Salud, Farmacorresistencia Microbiana, Educación, Eficacia, Encuestas y Cuestionarios

## Abstract

evaluar los elementos educativos y las acciones del Programa de Gestión de Antimicrobianos (PGA) en Unidades de Cuidados Intensivos Pediátricos (PED-UCI) en Brasil.

estudio multicéntrico y prospectivo, realizado entre octubre de 2022 y enero de 2023 mediante formulario electrónico. Se analizaron acciones educativas dirigidas a profesionales y pacientes sobre resistencia microbiana, además de intervenciones para mejorar la prescripción, como auditorías prospectivas, *feedback* y preautorización. Los datos se agruparon por ubicación, perfil asistencial y número de camas y se analizaron con métodos estadísticos paramétricos y no paramétricos.

de las 219 PED-UCI con PGA implementado, el 44,7% se ubicaba en la región Sudeste y el 58,9% realizaba educación continua para profesionales. Solo el 6,8 % promovía actividades educativas para pacientes y acompañantes, concentradas en hospitales públicos con más de 200 camas. El componente educativo mostró fragilidades, especialmente en hospitales más pequeños, mientras que las acciones para mejorar la prescripción estuvieron más consolidadas, con un 70,8% de implementación. Los hospitales privados y los de las regiones Sudeste y Sur destacaron en la adhesión a las prácticas de gestión.

el estudio reveló disparidades significativas en la implementación de los PGA en PED-UCI brasileñas, evidenciando la necesidad de reforzar la educación continua e involucrar a todos los actores para ampliar la adhesión, reducir la resistencia antimicrobiana y mejorar la atención pediátrica.

## Destacados

**(1)** El PGA en las PED-UCI brasileñas muestra lagunas en educación y prescripción antimicrobiana. **(2)** La educación insuficiente compromete el uso racional de antimicrobianos en Brasil. **(3)** La desigualdad regional desafía al PGA en hospitales públicos y privados. **(4)** Los hospitales de mayor tamaño y privados lideran la adhesión al PGA en las PED-UCI de Brasil. **(5)** Las estrategias educativas y las auditorías fortalecen el control antimicrobiano en el PGA.

## Introducción

La resistencia a los antimicrobianos (AMR) es una amenaza global para la salud pública, especialmente en América Latina, donde ha aumentado significativamente en los últimos años^(1-2)^. Una estrategia para seguir esta situación consiste en comprender el desarrollo de los Programas de Gestión de Antimicrobianos (PGA) y las barreras para su implementación, garantizando acciones y financiamiento para una ejecución eficaz y la sostenibilidad del programa^(3-4)^.

La Organización Mundial de la Salud (OMS) delineó un plan de acción global para abordar la AMR, destacando el papel fundamental de los *Antimicrobial Stewardship Programs* (ASP) en el afrontamiento de este problema^(3)^.

En Brasil, los esfuerzos comenzaron en 1998 y culminaron con la elaboración de planes de acción nacionales contra la AMR y directrices para la implantación del PGA. En 2019 se realizó la primera evaluación nacional en hospitales con Unidades de Cuidados Intensivos de Adultos (AD-UCI)^(5-8)^. Este proceso se amplió con la segunda encuesta (2022/2023), que incluyó hospitales con y sin AD-UCI y evaluó también las Unidades de Cuidados Intensivos Pediátricas (PED-UCI).

El PGA es una estrategia diseñada para implementar medidas que prevengan daños relacionados con el uso inadecuado de antimicrobianos, una de las principales causas de eventos adversos medicamentosos en el ámbito sanitario^(9)^. Mejorar los desenlaces clínicos, como morbilidad y mortalidad, y optimizar los costos son objetivos centrales del programa^(3)^. Para ello, es fundamental la colaboración de todos los profesionales implicados, basada en comportamientos positivos y un liderazgo clínico eficaz^(10-11)^.

La población pediátrica está cada vez más expuesta, especialmente en las PED-UCI, donde existe escasez de información confiable y objetiva sobre AMR y PGA en estos entornos asistenciales^(1,12-13)^.

Existen lagunas significativas de conocimiento en el manejo de antimicrobianos, agravadas por dificultades técnicas como la escasez de profesionales cualificados y la ausencia de estrategias educativas robustas y sistemáticas para la capacitación continua^(11,14-15)^. Estas lagunas comprometen el desarrollo y la implementación eficaz de acciones para mejorar la prescripción de antimicrobianos, que exigen no solo conocimiento técnico, sino también la integración de aspectos como indicación, prescripción, dispensación, dilución, administración, reevaluación continua y monitorización de reacciones adversas y evolución clínica. Estos elementos son cruciales para garantizar el uso racional de antimicrobianos y para la incorporación de datos reales en programas institucionales de educación y control. La falta de alineación entre las etapas del ciclo del medicamento y los programas educativos institucionales refleja desafíos estructurales y operativos que demandan estrategias más consistentes para la capacitación multiprofesional y para el fortalecimiento de las acciones de monitorización y auditoría^(6)^.

Se destaca además la dificultad real de medir el uso de antimicrobianos en pediatría^(16)^. Una forma útil de evaluación, especialmente en niños hospitalizados, es el cálculo de días de terapia (DOT) o días libres de terapia (AFD)^(6,17)^. Comprender la evolución de la AMR sigue siendo un desafío global, especialmente en la atención pediátrica, a menudo desatendida.

El objetivo de este estudio fue evaluar los elementos educativos y las acciones del Programa de Gestión de Antimicrobianos (PGA) en Unidades de Cuidados Intensivos Pediátricos (PED-UCI) en Brasil.

## Método

### Diseño del estudio

Se trata de un estudio prospectivo, transversal y multicéntrico.

### Lugar

El estudio se realizó en hospitales con unidades de cuidados intensivos pediátricos (PED-UCI) situados en distintas regiones de Brasil, abarcando instituciones públicas y privadas.

### Período

Los datos se recopilaron entre octubre de 2022 y enero de 2023.

### Población

Según datos de la *Agência Nacional de Vigilância Sanitária* (ANVISA), en 2022 Brasil contaba con 662 hospitales con PED-UCI distribuidos por las regiones. La región Sudeste se destacó con 346 hospitales (52,26%), seguida por las regiones Nordeste con 110 (16,62%) y Sur con 83 (12,54%). La población del estudio estuvo compuesta por hospitales brasileños con PED-UCI que poseían programas formalizados de PGA. Participaron en la encuesta 393 PED-UCI (59,36%), de las cuales 219 tenían PGA implementado y conformaron la muestra de este estudio^(5-8)^.

### Criterio de selección

Se incluyeron en el estudio los hospitales que disponían de unidades de cuidados intensivos pediátricos (PED-UCI) y que tenían PGA implantado.

### Definición de la muestra/Participantes

Se reclutaron los datos de los participantes del segundo sondeo nacional de PGA, coordinado por ANVISA/Ministerio de Salud, que incluyó hospitales de los tres niveles de gestión sanitaria (federal, estatal y municipal). El formulario fue completado por los servicios de salud tras el envío de correos electrónicos de ANVISA invitando a las instituciones a participar. La selección se basó en la adhesión voluntaria al instrumento de investigación por los profesionales representantes del PGA. La muestra quedó conformada por 219 hospitales con PED-UCI, representando instituciones de distintos tamaños y regiones de Brasil.

### Instrumento utilizado para la recolección de datos

La participación fue voluntaria, con el llenado de un formulario electrónico (*Google Forms* ^®^) por los profesionales representantes del PGA. Los datos se obtuvieron de un recorte de información de una base de una investigación multicéntrica. Se empleó un cuestionario estructurado, validado previamente, para evaluar las prácticas del PGA. Incluía cuestiones sobre las variables del estudio, distribuidas en secciones que abordaban educación, acciones de prescripción antimicrobiana y características institucionales^(18)^. La recolección se realizó mediante un sistema electrónico, garantizando mayor alcance y estandarización de las respuestas. El instrumento permitió clasificar los servicios en inadecuado, básico, intermedio o avanzado, según la puntuación otorgada a cada componente evaluado. Se aplicaron 46 preguntas dicotómicas (SÍ/NO) y 15 preguntas de respuesta múltiple.

### Recolección de datos

Los datos se recopilaron entre octubre de 2022 y enero de 2023, utilizando un instrumento validado para evaluar la implementación de los PGA^(18)^.

### Recolección de datos y variables del estudio

Para los fines de este estudio se realizó un recorte de los datos obtenidos y se examinaron solo los hospitales con PED-UCI que tenían PGA implementado y el componente 3: educación, y el componente 4: desarrollo de acciones para mejorar la prescripción de antimicrobianos. El componente 3 evaluó las acciones de formación dirigidas a los profesionales de la institución y a los pacientes sobre resistencia microbiana y uso de antimicrobianos. El componente 4 analizó intervenciones como auditoría prospectiva, *feedback* y preautorización de antimicrobianos^(6)^. Asimismo, se consideraron características de dichos hospitales, como ubicación, perfil asistencial y tamaño.

Los hospitales se clasificaron en Privado: administrado por entidad particular, benéfica o filantrópica; y Público: administrado por entidad gubernamental municipal, estatal o federal^(19)^. De forma conveniente, según el número de camas de atención, se agruparon en hasta 100 camas, 101/199 camas y ≥ 200 camas, y de acuerdo con las regiones brasileñas^(19)^.

### Tratamiento y análisis de los datos

Todos los análisis fueron realizados utilizando el *Statistical Package for the Social Sciences* (SPSS, v. 23, IBM, Armonk, NY). La comparación de las puntuaciones continuas del componente 3: educación y del componente 4: desarrollo de acciones para mejorar la prescripción de antimicrobianos en PED-UCI entre el tipo de institución, el número de camas disponibles y la región se efectuó mediante dos enfoques: el primero con pruebas paramétricas aplicando el procedimiento de *bootstrapping* con 5000 remuestreos y el método corregido y acelerado por sesgo (BCa - *Bias-corrected and accelerated*); y el segundo con pruebas no paramétricas debido a la distribución no normal de las variables.

Los resultados se expresaron en medidas de tendencia central (media y mediana) y de dispersión [desviación estándar (DE) e intervalo intercuartílico (IIC)], agrupados por tipo de atención y número de camas.

La comparación según el tipo de institución utilizó las pruebas t de Student para muestras independientes y U de Mann-Whitney, mientras que las comparaciones según el número de camas disponibles y la región emplearon el análisis de varianza (ANOVA) de un factor y la prueba de Kruskal-Wallis. Además, se realizaron pruebas a posteriori de Games-Howell para identificar qué pares diferían, ajustando los valores de p según el número de comparaciones entre grupos^(20)^. Las diferencias estandarizadas se calcularon mediante la medida de tamaño de efecto d de Cohen (d) para identificar la magnitud de la diferencia. Los valores de d se clasificaron conforme a los puntos de corte: d ≤ 0,49 = diferencia pequeña; 0,50 ≤ d ≤ 0,79 = diferencia media; y d ≥ 0,80 = diferencia grande.

Por último, las comparaciones de las frecuencias relativas de respuestas “sí” entre el tipo de institución, el número de camas disponibles y la región se realizaron mediante la prueba z a posteriori en el análisis de chi-cuadrado, ajustando los valores de p por el método de Bonferroni según el número de comparaciones entre grupos. Se consideraron estadísticamente significativos los valores de p ≤ 0,05^(20)^.

### Aspectos éticos

El estudio fue aprobado por el comité de ética en investigación con seres humanos de la *Universidade de Santa Cruz do Sul* (Certificado de Presentación para Apreciación Ética - CAAE: 57231722.3.1001.5343).

## Resultados

De los 219 hospitales con PED-UCI se identificaron 18 categorías profesionales que integran los equipos del PGA, destacándose el médico infectólogo, el representante de la Comisión de Control de Infecciones Hospitalarias, el farmacéutico clínico, el enfermero y el representante de la Comisión de Farmacia y Terapéutica.

El nivel de adhesión general del componente 3, relacionado con la educación, y del componente 4, que aborda el desarrollo de acciones para mejorar la prescripción de antimicrobianos en PED-UCI, fue el mismo para todas las regiones. El componente 3 alcanzó nivel básico, mientras que el componente 4 logró nivel avanzado, con un promedio de 155 (70,8%) acciones consolidadas.

De las instituciones que respondieron al estudio, el 44,7% se encontraba en la región Sudeste y el 22,4% en la región Sur. Más del 60,0% de los respondientes pertenecían a hospitales privados, con destaque para la región Sudeste (44,4%) y la región Sur (25,0%). En cuanto al número de camas hospitalarias, con excepción de la región Norte, todas las demás presentaron hospitales con más de 200 camas, observándose la mayor proporción en la región Sudeste (43,8%). Los datos relativos a estas categorizaciones se presentan en la Tabla 1.

**Tabla 1-:** Caracterización absoluta (n\*) y relativa (%^†^) por regiones, tipo de hospital y número de camas de los 219 hospitales que tenían PGA^‡^ en Brasil, encuesta de 2023. Santa Cruz do Sul, RS, Brasil, 2023

	**Tipo de hospital** **n** * **(%** ^†^ **)**		Total n* (% ^†^ )	**Número de camas** **n** * **(%** ^†^ **)**		
	**Privado**	**Público**		**Hasta 100 camas**	**101/199 camas**	**≥ 200 camas**
Norte	12 (63,2)	7 (36,8)	19 (100)	6 (31,6)	9 (47,4)	4 (21,1)
Nordeste	23 (63,9)	13 (36,1)	36 (100)	7 (19,4)	8 (22,2)	21 (58,3)
Centro Oeste	14 (82,4)	3 (17,6)	17 (100)	2 (11,8)	4 (23,5)	11 (64,7)
Sudeste	71 (72,4)	27 (27,6)	98 (100)	22 (22,4)	27 (27,6)	49 (50,0)
Sur	40 (81,6)	9 (18,4)	49 (100)	4 (8,2)	18 (36,7)	27 (55,1)
Total n (%)	160 (100)	59 (100)	219 (100)	41 (100)	66 (100)	112 (100)

*n = Número; ^†^% = Porcentaje; ^‡^PGA = Programa de Gestión de Antimicrobianos

La Tabla 2 muestra que no hubo diferencias en las puntuaciones mediante las pruebas paramétricas con procedimiento de *bootstrapping* (p > 0,05) entre hospitales públicos y privados, número de camas y regiones según el nivel de adhesión al PGA, en relación con los Componentes 3 (educación) y 4 (desarrollo de acciones para mejorar la prescripción de antimicrobianos), respectivamente.

**Tabla 2-:** Comparación de los componentes 3 (educación) y 4 (desarrollo de acciones para mejorar la prescripción de antimicrobianos) entre el tipo de institución, número de camas y región geográfica de los 219 hospitales que tenían PGA* en Brasil, encuesta de 2023. Santa Cruz do Sul, RS, Brasil, 2023

	**n** ^†^	**Componente 3**		***P*** ^||^	**Componente 4**		***P*** ^||^
		**Media ± DE** ^‡^	**Mediana (IIC)** ^§^		**Media ± DE** ^‡^	**Mediana (IIC)** ^§^	
Tipo de institución							
Privada	160	37,44 ± 36,42	40 (0; 65)	0,536	252,97 ± 53,50	262,5 (231,25; 285)	0,510
Pública	59	41,53 ± 43,49	45 (0; 70)		247,71 ± 51,80	255 (205; 290)	
Número de camas							0,276
Hasta 100	41	35,00 ± 33,37	40 (0;66,25)	0,768	241,10 ± 57,19	260 (225; 286,25)	
101 a 199	66	39,77 ± 42,29	42,5 (0; 70)	0,768	249,24 ± 52,76	265 (235; 293,75)	0,276
Más de 200	112	39,11 ± 37,95	40 (0; 65)		256,74 ± 51,32	260 (207,5; 280)	
Región							
Centro-Oeste	17	35,88 ± 40,59	35 (0; 62,5)	0,534	270,29 ± 35,11	275 (252,5; 290)	0,324
Nordeste	36	38,47 ± 34,90	42,5 (0; 70)		250,69 ± 67,11	275 (211,25; 303,75)	
Norte	19	59,21 ± 55,86	55 (0; 90)		251,05 ± 55,82	250 (225; 315)	
Sudeste	98	35,31 ± 36,42	37,5(0; 70)		250,56 ± 50,60	260 (223,75; 285)	
Sur	49	37,96 ± 34,82	45 (0; 60)		247,86 ± 50,71	260 (232,5; 280)	

*PGA = Programa de Gestión de Antimicrobianos; ^†^n = Número; ^‡^DE = Desviación estándar; ^§^IIC = Intervalo intercuartílico; ^||^P = Nivel de significación

Los resultados indican que los hospitales privados obtuvieron medias de 37,44 ± 36,42 en el Componente 3 y 252,97 ± 53,50 en el Componente 4, mientras que los públicos presentaron medias de 41,53 ± 43,49 y 247,71 ± 51,80, respectivamente. En cuanto al número de camas, los hospitales con hasta 100 camas presentaron medias de 35,00 ± 33,37 en el Componente 3 y 241,10 ± 57,19 en el Componente 4; aquellos con 101 a 199 camas, 39,77 ± 42,29 y 249,24 ± 52,76; y los con más de 200 camas, 39,11 ± 37,95 y 256,74 ± 51,32, respectivamente. Regionalmente, la mayor media en el Componente 4 se observó en la región Centro-Oeste (270,29 ± 35,11), mientras que en el Componente 3 la media fue de 35,88 ± 40,59.

La Figura 1 presenta las frecuencias absolutas de respuestas afirmativas (SÍ) en la encuesta relacionada con el Componente 3, que aborda actividades educativas sobre el uso racional de antimicrobianos. Estas respuestas se distribuyen según el tipo de hospital, número de camas y regiones geográficas. De los 219 hospitales participantes, 129 (58,9%) informaron la realización de alguna actividad educativa dirigida al tema. Los resultados destacan que los hospitales públicos registraron mayor frecuencia absoluta de respuestas afirmativas, especialmente en acciones destinadas a la educación de pacientes y acompañantes sobre el uso racional de antimicrobianos.

El análisis por tamaño hospitalario indica que los hospitales de tamaño medio, con 101 a 199 camas, presentaron mayor adhesión a las actividades educativas en comparación con otras categorías de tamaño. Regionalmente, la región Norte obtuvo la mayor frecuencia de actividades relacionadas con la educación sobre antimicrobianos, con destaque para los hospitales públicos de dicha región. Estos resultados evidencian la variación en la adhesión a las prácticas educativas entre los diferentes contextos institucionales y regionales, según lo detallado en la Figura 1.

**Figura 1-:** Comparación del comportamiento del componente 3 (educación) entre el tipo de institución, número de camas y región de los 219 hospitales que tenían PGA* en Brasil, según la encuesta de 2023. Santa Cruz do Sul, RS, Brasil, 2023

*PGA = Programa de Gestión de Antimicrobianos

La Figura 2 expresa el comportamiento de las acciones para mejorar la prescripción de antimicrobianos (componente 4). Las instituciones presentaban un 70,8% de acciones consolidadas. Se observó que la frecuencia absoluta (respuesta SÍ en la encuesta) se destacó en los hospitales privados, con más de 200 camas, de la región Sudeste de Brasil.

**Figura 2-:** Comparación del comportamiento del componente 4 (desarrollo de acciones para mejorar la prescripción de antimicrobianos) entre el tipo de institución, número de camas disponibles y región de los 219 hospitales que tenían PGA* en Brasil, encuesta de 2023. Santa Cruz do Sul, RS, Brasil, 2023

*PGA = Programa de Gestión de Antimicrobianos

Las instituciones privadas presentaron mayor adhesión a las acciones relacionadas con el Componente 4, destacándose prácticas como la revisión regular de prescripciones, la adopción de sistemas de apoyo a la toma de decisiones clínicas y la implementación de protocolos institucionales. Las instituciones públicas, aunque con menor frecuencia general, se destacaron en acciones específicas, como la integración con comités de control de infecciones hospitalarias y la revisión de datos microbiológicos como apoyo a la prescripción. En cuanto al número de camas, los hospitales con hasta 100 camas presentaron menor adhesión general a las acciones del Componente 4, mientras que los hospitales con 101 a 199 camas mostraron mayor frecuencia en acciones como la revisión de prescripciones y el uso de datos microbiológicos. Los hospitales con más de 200 camas registraron la mayor adhesión, destacándose por prácticas estructuradas como la integración con comités de control de infecciones hospitalarias y la revisión regular de prescripciones.

Regionalmente, la mayor adhesión a las acciones del Componente 4 se identificó en las regiones Sudeste y Sur, con destaque para prácticas como la implementación de protocolos de prescripción basados en evidencia, la revisión de prescripciones y la integración con comités de control de infecciones hospitalarias. Aun así, se observaron esfuerzos específicos relevantes en las regiones Centro-Oeste y Norte, evidenciando la variabilidad en la implementación de prácticas dirigidas a mejorar la prescripción de antimicrobianos entre las diferentes regiones de Brasil.

Estos resultados indican que las instituciones privadas, los hospitales de mayor tamaño y los situados en las regiones Sudeste y Sur concentran la mayoría de las acciones orientadas al desarrollo de prácticas para mejorar la prescripción de antimicrobianos.

La Figura 3 muestra las comparaciones entre hospitales públicos y privados de los componentes 3 y 4 con p < 0,05. En la comparación de las puntuaciones generales del componente 3: educación y del componente 4: desarrollo de acciones para mejorar la prescripción de antimicrobianos en PED-UCI, se observó que no hubo diferencias en las puntuaciones mediante las pruebas paramétricas con procedimiento de *bootstrapping* (todas con p ≥ 0,276) entre el tipo de institución, número de camas disponibles y región. Las pruebas no paramétricas tampoco identificaron diferencias en las comparaciones (todas con p ≥ 0,309). Sin embargo, las comparaciones par a par mostraron una diferencia estadísticamente significativa en la puntuación del componente 4 entre las regiones Centro-Oeste y Sudeste [Intervalo de Confianza (IC) del 95 % de la diferencia: −38,04; −0,33; d = 0,41] y entre Centro-Oeste y Sur (IC95 % de la diferencia: −43,97; −0,42; d = 0,46).

**Figura 3-:** Comparación de la frecuencia relativa de respuestas “sí” a las preguntas de los componentes 3 (educación) y 4 (desarrollo de acciones para mejorar la prescripción de antimicrobianos) según el tipo de institución, el número de camas disponibles y la pregunta 4.3.3.8: monitorización sérica de los antimicrobianos del componente 4, entre las regiones, de los 219 hospitales que tenían PGA* en Brasil, encuesta de 2023. Santa Cruz do Sul, RS, Brasil, 2023

Nota: las letras subíndices indican diferencias estadísticamente significativas (p de Bonferroni < 0,05) de frecuencias relativas entre grupos; *PGA = Programa de Gestión de Antimicrobianos

## Discusión

Este estudio representa la primera investigación nacional sobre los recursos en PGA, evaluando el panorama de la educación y las actividades para mejorar la prescripción de antimicrobianos en las PED-UCI de Brasil. Los resultados indicaron variabilidad en las prácticas, evidenciando fragilidades en el desarrollo de programas de educación permanente y disparidades regionales e institucionales, especialmente entre hospitales públicos y privados. La predominancia de hospitales privados con más de 200 camas en las regiones Sudeste y Sur, que concentran la mayor parte de la población y del Producto Interno Bruto (PIB) del país, refleja desigualdades históricas en infraestructura y recursos sanitarios^(21)^. Esta concentración contrasta con la mayor cantidad relativa de hospitales con PED-UCI en el Nordeste, pero con menor adhesión al llenado de la encuesta, y con una menor frecuencia de hospitales con PGA implantado en las regiones Norte y Centro-Oeste.

Los programas formales de PGA en hospitales públicos y con hasta 200 camas son limitados, amplificando desafíos estructurales ya identificados, como la cuantificación del uso de antimicrobianos pediátricos, la definición de medidas de eficacia y la falta de directrices locales basadas en evidencia^(1,16,22)^. En consonancia, un estudio en América Latina destacó barreras como la ausencia de financiación, la escasez de oportunidades de capacitación durante la jornada laboral y la carencia de temas aplicables a la implementación de ASP y métricas para el cambio de comportamiento^(4)^. Estos obstáculos, comunes en países de renta baja y media, parecen reflejarse en el panorama brasileño, donde el 40% de las instituciones informó no poseer actividades educativas sobre antimicrobianos.

La comparación entre los Componentes 3 (educación) y 4 (desarrollo de acciones) reveló discrepancias. Mientras que el Componente 4 mostró mayor consolidación, con el 70 % de las acciones implementadas, el Componente 3 evidenció lagunas significativas, especialmente en hospitales más pequeños y públicos. Este hallazgo es coherente con estudios internacionales que reportaron la ausencia de programas regulares de educación en países de renta baja y media^(17)^. La formación de profesionales y la educación permanente son herramientas esenciales para aumentar la comprensión de la resistencia antimicrobiana (AMR) y promover cambios de comportamiento^(23-25)^. La introducción de estrategias innovadoras, como sistemas rápidos de aprobación de antimicrobianos mediante mensajes de texto, redujo costos y optimizó el uso de medicamentos en estudios recientes^(26)^.

Los resultados también destacaron que la implementación de protocolos basados en evidencia, como los destinados a síndromes infecciosas específicas, estaba presente en el 89% de las instituciones, pero con frecuencia sin auditorías que garanticen su aplicación. La inexistencia de directrices prácticas para el uso racional de antimicrobianos se asoció al aumento del consumo de antibióticos de amplio espectro, en línea con estudios que identificaron una relación entre protocolos frágiles y mayor morbimortalidad infantil^(27-28)^.

El análisis de la Figura 3 refuerza la importancia de las intervenciones educativas y de la armonización de prácticas entre las diferentes regiones y tipos de hospitales. Por ejemplo, la monitorización sérica de antimicrobianos mostró diferencias estadísticamente significativas entre las regiones Centro-Oeste, Sudeste y Sur, evidenciando mayor adhesión en las regiones más desarrolladas. Este hallazgo corrobora estudios previos que indicaron la necesidad de adaptación local para optimizar la implementación de programas de PGA^(29-39)^. Además, la retroalimentación a los prescriptores tras las auditorías y la optimización de las dosis de antimicrobianos (farmacocinética/farmacodinámica) fueron más frecuentes en hospitales privados con más de 200 camas, reforzando la desigualdad estructural entre los sistemas público y privado.

En este contexto, se destaca el papel estratégico del equipo de enfermería. Al estar directamente involucrado en etapas críticas del cuidado, como la administración, monitorización y notificación de antimicrobianos, la enfermería debe ser el foco de acciones educativas permanentes que amplíen su protagonismo, su cualificación técnica y su actuación clínica en el ámbito de los PGAs. Además, considerando la centralidad del enfermero en la relación con los pacientes y sus familiares, especialmente en unidades pediátricas, es esencial que los programas educativos también contemplen la orientación continua a los acompañantes, promoviendo el uso racional de antimicrobianos y fortaleciendo la adhesión a las prácticas de prevención de la resistencia microbiana.

Paralelamente, los sistemas de registro de enfermería, a menudo subvalorados, necesitan ser mejorados, informatizados e integrados al PGA, permitiendo la trazabilidad de las acciones, el soporte a la auditoría clínica y la retroalimentación de datos. Esta integración entre la educación multiprofesional, la cualificación de la comunicación con los usuarios y el fortalecimiento de los registros puede contribuir decisivamente a la sostenibilidad y eficacia de los programas, especialmente en contextos pediátricos que demandan un cuidado altamente sensible y centrado en la familia.

Las limitaciones del estudio incluyen el riesgo de sesgo de selección, debido a la participación voluntaria de los hospitales, y las diferencias en la frecuencia de respuestas entre las regiones, lo que puede no reflejar el panorama general de Brasil. Sin embargo, el estudio aporta datos inéditos y relevantes para guiar políticas e intervenciones institucionales que favorezcan la adhesión al PGA, principalmente en escenarios pediátricos. La integración de estrategias educativas y de control de infecciones con apoyo multiprofesional puede contribuir a la reducción del uso excesivo de antimicrobianos y a la mejora de los indicadores de morbimortalidad infantil.

## Conclusión

Los resultados de esta encuesta ponen de relieve un desequilibrio significativo entre los distintos servicios hospitalarios de Brasil, incluidos hospitales públicos y privados con hasta 100 camas, entre 101 y 199 camas y más de 200 camas, además de las disparidades regionales en cuanto a los recursos educativos disponibles para profesionales, pacientes y acompañantes, así como en las acciones destinadas a mejorar la prescripción de antimicrobianos en el PGA. Esta variabilidad refleja desigualdades estructurales y geográficas que exigen un enfoque nacional integrado y colaborativo para combatir la AMR.

Una de las principales prioridades identificadas en este estudio fue la necesidad urgente de educación permanente, tanto para los profesionales de la salud como para los usuarios del servicio. Los programas educativos sistemáticos, continuos y respaldados por gestiones comprometidas con el PGA son fundamentales para sostener cambios de comportamiento y promover el uso criterioso de antimicrobianos. Esta brecha educativa constituye un reto crítico, especialmente en los hospitales más pequeños, pero también una oportunidad de impacto significativo.

En este escenario, se destaca la necesidad de reposicionar la enfermería como agente articulador entre la gestión del cuidado, la educación en salud y la seguridad del paciente. La inserción cualificada del equipo de enfermería en los PGA puede ampliar el alcance de las acciones educativas dirigidas a profesionales, pacientes y familias, además de contribuir a la integración de las prácticas clínicas con los sistemas de registro, monitoreo y auditoría. Invertir en la capacitación técnica y relacional de estos profesionales, así como en la valorización de su actuación en los registros asistenciales, representa una estrategia viable y de alto impacto para consolidar el uso racional de antimicrobianos en entornos pediátricos críticos. Al fortalecer estas dimensiones, Brasil podrá no solo reducir desigualdades estructurales en la lucha contra la resistencia microbiana, sino también cualificar la atención y promover avances sostenibles en la salud infantil.

Dado el historial de éxito de Brasil en programas de salud pública, como el Programa Nacional de Vacunación Infantil, es posible replicar estrategias similares para fortalecer la adhesión al PGA y reducir la morbimortalidad asociada a la AMR en las PED-UCI. Un próximo paso esencial será realizar análisis de costo-efectividad de las intervenciones propuestas, incluido el monitoreo del perfil de microorganismos causantes de infecciones y del uso de antimicrobianos, lo que permitirá una planificación más estratégica y eficiente.

Los esfuerzos coordinados gubernamentales y no gubernamentales serán indispensables para afrontar este desafío. Tales esfuerzos deben incluir la ampliación del acceso a medicamentos de calidad, la mejora de la infraestructura hospitalaria y sanitaria, la expansión logística de la microbiología clínica y el monitoreo adecuado de las infecciones. Además, las organizaciones profesionales tienen un papel esencial al facilitar redes de colaboración nacionales e internacionales para compartir buenas prácticas, capacitar profesionales y apoyar la implementación de estrategias adaptadas a las realidades locales.

Esa estructura colaborativa puede aumentar la eficacia de las acciones de vigilancia y utilización de antimicrobianos, fortaleciendo los indicadores de control de la resistencia antimicrobiana y reduciendo el impacto global de la AMR en niños. El fortalecimiento del PGA en Brasil no es solo una necesidad, sino una oportunidad para promover avances sostenibles y significativos en la salud infantil y en la seguridad de los servicios de salud pediátrica.
